# The functional role of serial dependence

**DOI:** 10.1098/rspb.2018.1722

**Published:** 2018-10-31

**Authors:** Guido Marco Cicchini, Kyriaki Mikellidou, David C. Burr

**Affiliations:** 1 Institute of Neuroscience, National Research Council (CNR), Pisa, Italy; 2 Department of Translational Research on New Technologies in Medicine and Surgery, University of Pisa, Pisa, Italy; 3 Department of Psychology, University of Cyprus, Nicosia, Cyprus; 4 Department of Neuroscience, University of Florence, Florence, Italy; 5 School of Psychology, University of Sydney, Sydney, Australia

**Keywords:** serial dependence, Kalman filter, Bayesian, optimal behaviour, orientation

## Abstract

The world tends to be stable from moment to moment, leading to strong serial correlations in natural scenes. As similar stimuli usually require similar behavioural responses, it is highly likely that the brain has developed strategies to leverage these regularities. A good deal of recent psychophysical evidence is beginning to show that the brain is sensitive to serial correlations, causing strong drifts in observer responses towards previously seen stimuli. However, it is still not clear that this tendency leads to a functional advantage. Here, we test a formal model of optimal serial dependence and show that as predicted, serial dependence in an orientation reproduction task is dependent on current stimulus reliability, with less precise stimuli, such as low spatial frequency oblique Gabors, exhibiting the strongest effects. We also show that serial dependence depends on the similarity between two successive stimuli, again consistent with the behaviour of an ideal observer aiming at minimizing reproduction errors. Lastly, we show that serial dependence leads to faster response times, indicating that the benefits of serial integration go beyond reproduction error. Overall our data show that serial dependence has a beneficial role at various levels of perception, consistent with the idea that the brain exploits the temporal redundancy of the visual scene as an optimization strategy.

## 1. Introduction

As most objects in the environment are relatively stable over time, there are large temporal redundancies in the spatio-temporal flow of information. It has long been known that sensory systems exploit spatial redundancies by shifting their responses to match the stimulation statistics [1], but until recently there has been little evidence as to whether perceptual systems carried over information across time.

Two recent papers [2,3] introduced a new psychophysical paradigm, *serial dependence*, which provided direct evidence of how a system incorporates past information into the perception of the current stimulus. These effects have now been confirmed with a variety of stimuli and tasks, from simple orientation judgements [3–5], numerosity [2], position [6,7], facial identity and expression [8,9], eye gaze [10], pulchritude [11] or body size [12], to complex judgements such as summary statistics [13], variance [14] and confidence [15]. A series of control experiments showed that serial dependence effects could not be accounted for by effects such as priming, hysteresis, explicit memory or expectation. Furthermore, functional magnetic resonance imaging results [16] have shown that neural representations in the primary visual cortex (V1) were biased towards previous perceptual decisions, demonstrating a direct neural correlate of serial dependence, and suggesting that the effects occur early in primary visual cortex.

In non-symbolic numerosity judgements, serial dependence effects were found to be strong enough to compress the subjective spatial representation of numbers [2], an effect previously thought to reflect the logarithmic encoding of numbers [17]. The compression is a direct result of the fact that not all stimuli have the same level of dependence on the previous presentation: low numerosities, which are reproduced more reliably (with less variability) than higher numerosities, showed less serial dependence. This suggests that serial dependence is related to the *reliability* of the current sensory information, which prompted a model where serial dependence becomes a form of response optimization.

Here we develop an ideal observer model and test its predictions with an orientation reproduction task. To this aim, we first test if serial dependence is stronger with less reliable stimuli, varying reliability by varying the orientation and spatial frequency of grating patches [18,19]. We then explore how serial dependence changes as a function of inter-stimulus orientation change. We also show that besides reducing error, serial dependence leads to faster responses. All this evidence shows that serial dependence increases the accrual of sensory information, improving efficiency.

## 2. Methods

### (a) General procedure

The study was approved by the Regional Ethics Committee (Comitato Etico Pediatrico Regionale-Azienda Ospedaliero Universitaria Meyer-Firenze) and was in accordance with the ethical standards of the 1964 Declaration of Helsinki. Informed written consent was obtained from each participant prior to the experiments. Ten participants (two authors plus four naive observers for experiment 1, two authors plus four naive for experiment 2; mean age = 31, range = 28–41), all with normal or corrected-to-normal vision participated in the study.

Stimuli were presented on the face of a calibrated 23 inch LCD monitor subtending 26° (horizontal) by 14.5°. Stimuli were generated using MATLAB (the MathWorks, Natick, MA) in conjunction with routines from the Psychtoolbox [20]. Responses were collected via a standard mouse and keyboard connected via USB to a PC yielding a temporal resolution of 4 ms.

### (b) Experiment 1

Experiment 1 investigates the effect of stimulus reliability on serial dependence by manipulating the orientation and spatial frequency of the Gabor patch in a 2 × 2 design. Stimulus orientation was either oblique (close to the diagonal: 25°–65° in steps of 10°) or cardinal (close to vertical: −20° to +20°, in steps of 10°). The spatial frequency of the Gabor also varied; either 0.3 or 1.2 cycles per degree (cpd). Following Fischer & Whitney [3], the spatial frequency content of the mask was matched to that of the stimulus.

**Figure 1.**
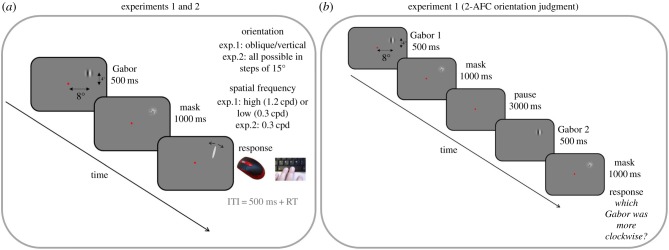
Timeline and stimuli. (*a*) In experiments 1 and 2 we presented a Gabor stimulus for 500 ms, followed by a mask for 1000 ms and a response oval which the participants had to adjust in order to match the orientation of the Gabor and then confirming their response by pressing the spacebar of the keyboard. In experiment 1, the orientation and spatial frequency (SF) of the Gabor were manipulated in a 2 × 2 design. Stimulus orientation in ‘oblique’ conditions was close to the diagonal (from 25° to 65°, in steps of 10°), whereas in ‘cardinal’ conditions it was close to vertical (−20° to + 20°, in steps of 10°). The spatial frequency of the Gabor was either 0.3 or 1.2 cycles per degree (cpd) in ‘low SF’ or ‘high SF’ conditions respectively. In experiment 2, the orientation of the Gabor was all around the clock in steps of 15° and the spatial frequency was fixed at 0.3 cpd. (b) In experiment 1 in separate sessions we asked a 2-AFC orientation judgement task, of two Gabors followed by a mask and separated by 3000 ms; participants were asked to indicate which was more clockwise. (Online version in colour.)

The experimental paradigm (figure 1*a*) was a close replication of the adjustment paradigms of Fischer & Whitney [3]. Each trial began with the presentation of an eccentric Gabor stimulus (contrast 25%, 500 ms, 3.2° full-width half-height), followed by a mask (random noise filtered, contrast 50%, 1000 ms) rightward of fixation (8° horizontal, 4° vertical eccentricity). Participants were instructed to reproduce the orientation of the Gabor patch by moving the mouse and setting the orientation of an oval (width 0.2°, length 1°). Participants confirmed the orientation with the space bar of the keyboard and the reproduction bar disappeared.

We expressed the strength of serial dependence as the weight of the previous orientation on the current judgement (figure 3*e–h*). To combine various conditions, we plotted reproduction bias (current reproduced orientation minus current stimulus orientation) on the ordinate against the orientation change across trials (orientation of the previous minus orientation of the current stimulus) on the abscissa. We excluded responses more than 3.5 s after the disappearance of the Gabor patch and also those deviating more than 30° from the physical orientation of the patch. A simple linear fit provides an estimate of the loading of previous orientation on current error. As serial effects are predicted and found to be maximal for relatively small stimulus differences [3,5] (see equation (3.1)), we restricted our linear fitting to trial pairs where the orientation changed between the previous stimulus and the current response was between −10° and +10°. Six subjects took part in the experiment each contributing 280 trials to each condition.

The same participants also performed a two-alternative forced choice (2-AFC) orientation judgement task to measure individual sensitivity (figure 1*b*). To mimic the typical serial dependence paradigm, we presented Gabors sequentially, so that only a single stimulus was present at a given time. Stimulus parameters, position and mask were the same as in the reproduction task. The two presentations were separated by a 3 s pause, which was the average temporal separation of stimuli in the serial dependence studies. Proportion of ‘more clockwise’ responses (of 80 trials participant^−1^) were plotted as a function of orientation difference between the first and the second stimulus to yield psychometric functions, which were fitted with cumulative Gaussians. The standard deviation (*σ*) of these functions is an estimate of the underlying noise distribution. As there were two stimulus presentations in each trial, the final estimate of reliability was given by given by *σ/√*2.

### (c) Experiment 2

Experiment 2 studied how serial dependence varies with similarity of stimuli. We presented all possible orientations in steps of 15° keeping spatial frequency fixed at 0.3 cpd (figure 1*a*), with all other parameters and timings the same as in experiment 1. Data were arranged in a two-dimensional space according to the orientation of the current and previous stimulus. For each combination of the two values, we calculated the average bias, the average root-mean-square error and average response time. To gain power in the analysis, we then averaged together the conditions with the same ‘previous-current’ orientation difference. By convention positive difference indicates that the previous trial was more clockwise than the current. Six participants completed the experiment leading to about 12 000 trials in total.

## 3. Results

### (a) Ideal observer model

Here we develop a model predicting how serial dependence can lead to optimal performance, gauged by measuring the deviation from correct responses in the face of sensory noise. The literature on multisensory integration and regression to the mean suggests that any sensory representation which can be characterized by noise can benefit when other information is taken into account [21–25]. Similarly, our model is essentially a weighted sum of the current and previous stimulus, to reduce noisiness:3.1
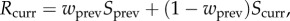
where *R*_curr_ is the response to the current stimulus. Current and previous stimuli are *S*_curr_ and *S*_prev_, the weight of the previous stimulus is *w*_prev_.

**Figure 2.**
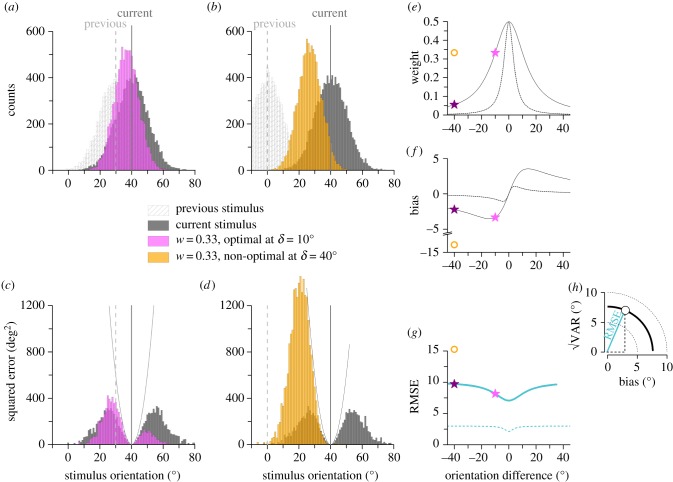
Optimal integration of current and previous sensory information. (*a–d*) Example of sensory representation of a 40° stimulus accompanied by a 10° estimation noise. This signal can either be integrated with a preceding stimulus of similar (*a,c*) or different (*b,d*) orientation (10° and 40° away respectively). (*a,c*) show representation histograms. Along with the representation of sensory estimates, we show the histograms of estimates provided by an observer who integrates current and previous signals with a 0.33 weight of the past (which is optimal for the 10° difference and non-optimal for the 40°difference). (*b,d*) show squared error distributions associated with each estimate of the observer: squared error grows fast for estimates which are far from the correct value (40°). In the case of small difference and optimal weight (*b*, pink) the observer performing integration fares better than the original set of estimates. In (*e*) we show how the weight of the previous stimulus should vary as function of distance between the two stimuli, for noise of 10° (continuous line) and 3° (dashed line). Panels (*f,g*) display the bias and root-mean-square error (RMSE) of the ideal observer employing optimal weighting of current and previous information. Again, two examples are shown assuming noise of 10° (continuous line) and 3° (dashed line). The conditions depicted in panels (*a–d*) are highlighted with a pink star (optimal weight for 10° difference) or a hollow circle (non-optimal weight with 40° difference) along with optimal choice for 40° (purple star). Inset (*h*) illustrates that RMSE is the Pythagorean sum of bias and the square root of variance.

In general, when an observer combines two signals there is a reduction of uncertainty as the overall variance is3.2
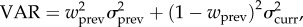
which is smaller than either of the two variances alone. At the same time, a linear combination may introduce a detrimental biasing term:3.3
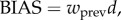
which is proportional to the weight of the other cue and the distance between the two cues. Overall the total squared error is given by their sum (figure 2*h*):3.4
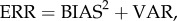
optimization entails the minimization of this quantity by selecting the appropriate *w*:
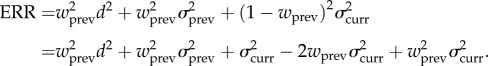

Rearranging so to highlight *w*_prev_ (the term to marginalize) yields3.5
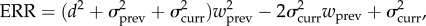
which is a quadratic function of *w*_prev_ (i.e. 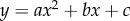) which is minimized when3.6
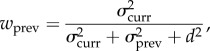


(i.e. for *x* = −*b*/2*a*).

When reliabilities of previous and current stimuli are the same, this can be simplified to3.7
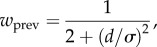
which reveals that the crucial variable is the ratio between the stimulus change and the sensory resolution.

The core idea of the model is illustrated in the example scenarios of figure 2*a–d*. The leftmost column shows hypothetical distributions of sensory representations of a currently displayed stimulus orientated at 40°, for which the resolution variability is 10° (figure 2*a*, grey). This information could in principle be used either alone or in conjunction with that from the previous stimulus which, in this example, was 10° away (mean = 30°, s.d. = 10°). If the observer uses the optimal weight (*w* = 0.33), the combined distributions of response estimates (given by the weighted product of the two original distributions) are slightly off the correct value, but also narrower (pink distribution in figure 2*a*). The lower plot (figure 2*c*) shows the distribution of overall squared error, calculated as the product of the magnitude of the error and the probability of its occurrence (figure 2*a*, grey). If the current sensory representation is used on its own (grey), the error distribution will be symmetrically bimodal. However, if the previous stimulus is combined in an optimal manner, with a weighting of 0.33 (pink distribution of figure 2*c*), the error distribution will become asymmetric, shifting to the left; but it will also become narrower, so the overall error (given by the area under the curve) is less than for the grey curve.

An important aspect of the model is that the weight given to the previous representation should be scaled down as the difference between past and present stimuli (*d* of equation (2.7)) increases. To illustrate this, we simulate a condition where a very different previous stimulus (40° more tilted) is combined with the present with an inappropriately high weighting of 0.33. The response distribution for the combined information is similarly narrower than either distribution alone (yellow distribution in figure 2*b*). However, the bias is now much larger (about 13° instead of 3°), and it produces squared error distributions that are very high, far higher than that for just considering the current stimulus. Clearly, a weighting of 0.33 for such large inter-stimulus distances is not optimal. Indeed, our formula prescribes that for 40° difference, the weight of the past should only be 0.056 (purple star in figure 2*e–g*).

Figure 2*e* shows the optimal weight as a function of orientation difference. The theoretical considerations above suggest that for very small differences the weight should be 0.5 (equal for past and present), then roll off as stimulus distance increases. Figure 2*f* shows the corresponding biasing errors, which are reminiscent of those found by Fischer & Whitney [3]. Figure 2*g* shows the overall root-mean-square error (RMSE) as function of stimulus distance, with the best performance obtained when two successive stimuli are identical, and thus serial effects are maximal. Many of these signatures are evident in published data. Here, we aim to test directly various aspects of the model, showing that serial dependence leads to optimizing perception.

### (b) Experiment 1

**Figure 3.**
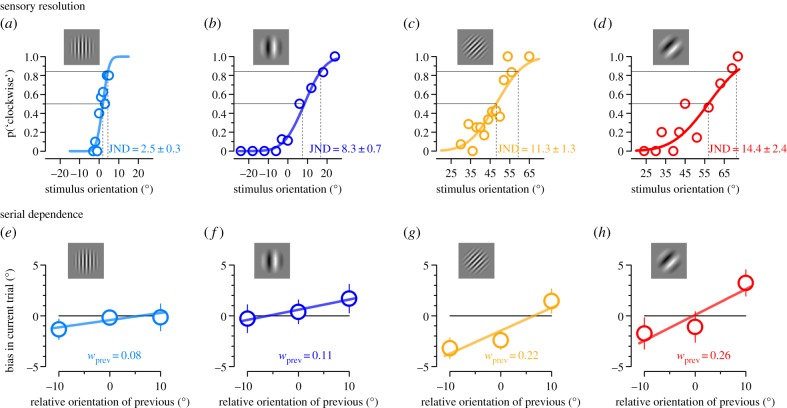
Orientation discrimination and serial dependence for gratings of different base orientation and spatial frequency. (*a–d*) Psychometric curves for discriminating which of two Gabors was more clockwise as function of the orientation of the second stimulus. The dashed vertical lines indicate 1 standard deviation, which we take as the estimate of just-noticeable difference (JND). (*e–h*) Bias in current trial induced by the relative orientation of the previous stimulus. The slope of the curve returns the loading of previous stimulus orientation on the current judgement and indicates serial dependence, indicated as weight. Cartoon of the stimuli (low or high spatial frequency, cardinal or oblique) are shown in insets.

We measured sensitivity and serial dependence in orientation judgements for four types of Gabor stimuli differing in average orientation (oblique, cardinal) and spatial frequency (low, high). Figure 3*a–d* shows psychometric functions for discriminating the orientation of the four types of stimuli. It is clear that the steepness of the psychometric functions depends on both spatial frequency and orientation: they are steeper (implying higher sensitivity) for cardinal stimuli of high spatial frequency, and shallower for oblique stimuli of low spatial frequency. Average just-noticeable differences (JNDs) (given by 1 s.d. of the cumulative Gaussian fit) are given in figure 3*a–d*. A two-way repeated measures ANOVA confirmed that high spatial frequencies yield lower thresholds than low spatial frequencies (*F*_1,5_ = 67.8, *p* = *0.0004*) and that cardinal stimuli yield lower thresholds than oblique stimuli (*F*_1,5_ = 21.3, *p* = *0.006*). No significant interaction was found (*F*_1,5_ = 1.34, *p* = *0.30*).

Figure 3*e–h* shows serial dependence for the four types of stimuli. In all cases, the current trial was biased towards the orientation of the previous trial: positive when it was positive, and negative when negative. We calculate the weights of the past stimuli by the slope of the best-fitting linear regression to the three data points. The estimated weights (shown in figure 3*e–h*) increase orderly from weakest serial dependence for high spatial frequency cardinal Gabors to highest effects with the low spatial frequency oblique Gabors. A two-way repeated measures ANOVA shows a main effect both of spatial frequency (*F*_1,5_ = 9.4, *p* = *0.028*) and average orientation (*F*_1,5_ = 7.2, *p* = *0.044*). No significant interaction was found (*F*_1,5_ = 0.001, *p* = *0.97*). This indicates that both factors have a strong and independent effect upon serial dependence.

**Figure 4.**
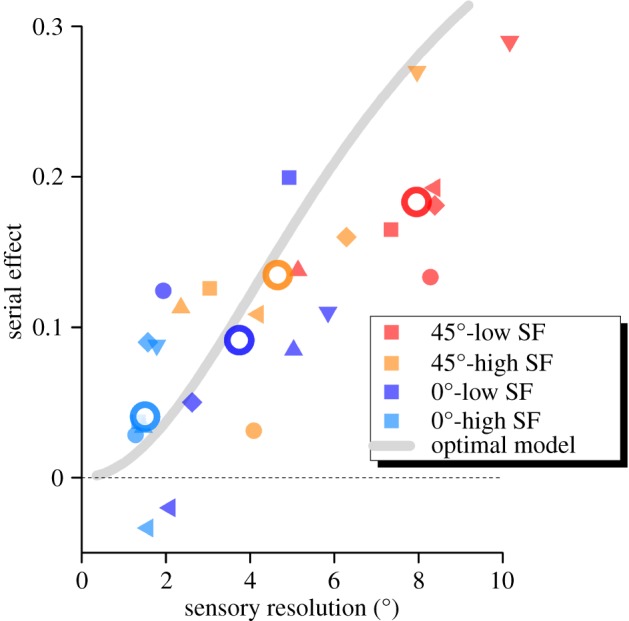
Strength of serial dependencies against JND in a 2-AFC orientation discrimination with four kinds of orientation Gabors (see colour code), for six observers (indicated by different symbol shape). The open circles show average data. The optimal model from equation (3.7) (with zero parameters) predicts well the relationship between serial dependencies and sensory discrimination.

To better explore the relationship between serial effects and sensory thresholds, we plot the strength of the serial effect against the discrimination threshold for all conditions and participants (figure 4). Superimposed on the data is the prediction of the ideal observer model of equation (3.1), which aims to minimize reproduction errors, considering both sensory noisiness and inter-stimulus distance. Note that there are no free parameters in this simulation, yet it follows the trend of the data very well (*R*^2^ = 0.21). If we allow a simple scaling factor of *k* = 0.75 (which could reflect underestimation by the system of its noisiness, or corruption of the memory trace), the fit improves to *R*^2^ = 0.58.

### (c) Experiment 2

**Figure 5.**
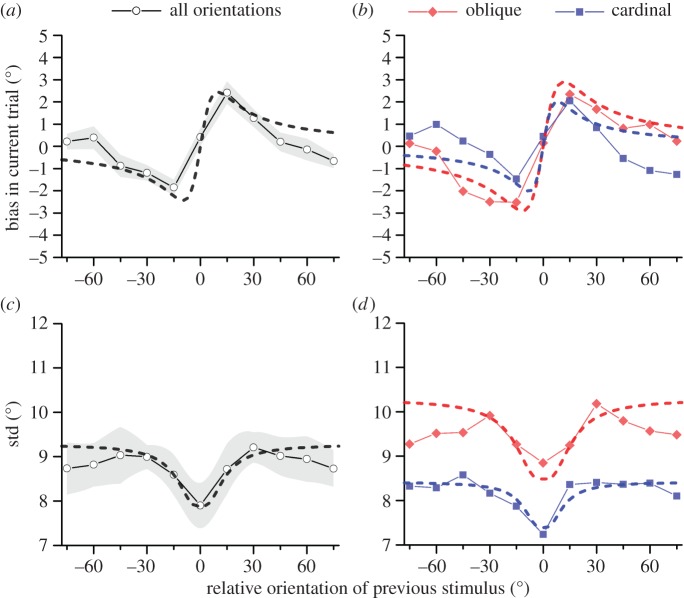
Bias and response scatter (std) in reproduction as function of orientation difference between previous and current stimulus. (*a,b*) Bias in current response as a function of the clockwise orientation difference between the previous and current stimulus. Positive values on the abscissa indicate that the previous trial was more clockwise than the present trial, and positive errors indicate that the reported orientation was more clockwise than the true stimulus. Different colours code different orientations (black, all orientations; red, orientations close to oblique; blue, orientations close to cardinal). Connected data points are average across six observers. Dashed lines are predictions of ideal observer with three levels of sensory noise (5.6, 6.7 and 8.3 corresponding to the average sensory resolution in the various stimulus ranges). (*c,d*) Average response scatter as a function of orientation difference between previous and current trial. The functions dip near zero indicating that when the current and previous stimuli were identical, responses are less scattered. All the other conventions as above. The ideal observer model has been complemented with a response stage which introduces 6° of motor noise.

Fischer & Whitney's initial report [3] showed that serial dependence is strongest when two successive stimuli are relatively similar. This fact is well captured by our ideal observer model, with the term *d^2^*on the denominator. To test further whether the model could predict this behaviour quantitatively, we measured serial dependence with low spatial frequency Gabors (which yield largest serial dependence effects) at all possible orientations in steps of 15°. Figure 5*a* shows the signed error in the current trial as a function of orientation difference between the previous stimulus and the current response. As reported by Fischer & Whitney [3], the maximum bias occurs at about 15° and scales down when stimulus differences are larger. The black curve shows the prediction of our ideal observer model to the average data across all orientations. Since in this experiment we did not collect independent measures of sensory resolution we employed the average sensory resolution for low spatial frequency stimuli in the previous experiment (6.7°). It is clear how the model provides a good match to the data, in particular in the central region (from –60 to +60°), where the fit is very good fit (*R*^2^ = 0.75).

The Bayesian-based ideal observer model reduces overall error in discriminating noisy sensory inputs [22,24,26] by averaging successive noisy stimuli (figure 2). Figure 5*c* plots the average response scatter (root variance) as a function of the difference in orientation between the current and previous stimuli. When two orientations are identical (*d* *=* *0*), responses are less scattered, about 15% less than when stimuli are 15°, 30° or 45° different (worst *t*-test is −15 versus 0 *t*_5_ = −2.6 *p* < 0.025). This is a clear signature of automatic averaging effects in the perceptual system. The black curve in figure 5*c* shows the predicted scatter of the ideal observer model, using the model employed in figure 5*a* (again assuming that the average sensory resolution is 6.7°), with the only extra assumption that the response adds a constant noise to all of the trials (adjusted to best-fit, about 6.2°). The model, with only one degree of freedom, captures well the pattern of data (*R*^2^ = 0.74). Figure 5*d* plots scatter independently for near cardinal and near oblique stimuli. As expected, the scatter in this condition inherits the amount of perceptual noise associated with each stimulus and near oblique stimuli have more response scatter. Again, the ideal observer (dashed lines), with sensory resolutions of *σ* = 5.6 and 8.2° for cardinal and oblique, and 6.2° of motor noise fixed from the fit of figure 5*c* provides a very good description of the data (*R*^2^ = 0.77 and *R*^2^ = 0.81 for oblique and cardinal).

**Figure 6.**
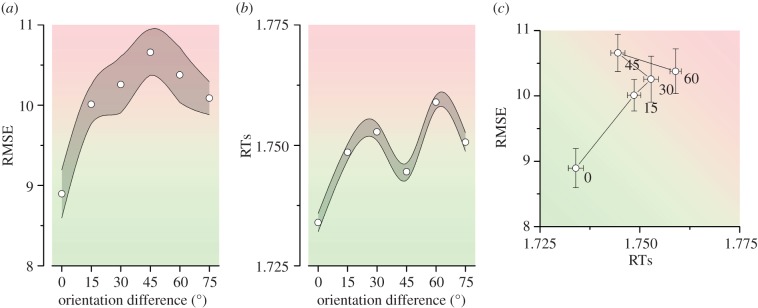
Behavioural advantage of serial dependence. (*a*) Root-mean square error (RMSE) of aggregate observer as function of absolute orientation difference between two successive trials. (*b*) Median response time (RT) of aggregate observer as function of absolute orientation difference between two successive trials. (*c*) Correlation between RMSE and RT. For identical stimuli, there is an advantage along both dimensions.

Figure 6*a* plots root mean-square error (RMSE), or total error in the reproduction responses as a function of the stimulus orientation difference between trials. RMSE is given by the Pythagorean sum of biasing errors and scatter errors, displayed independently in figure 5*a,c* (see illustration in figure 2*h* and equation (3.4)). This plot demonstrates the optimality of the model and the observer responses. Consistently with the predictions of the ideal observer, when two successive stimuli are identical (*d* = 0), serial dependencies should be at their highest yielding minimal error, which is what is found.

Although we did not ask subjects to make speeded responses, it is possible that the conditions which resulted in less error were accompanied by longer response times (speed-accuracy trade-off). We therefore plot median response times in figure 6*b*. The response times show a clear minimum for identical successive stimuli and increasing with orientation difference (figure 6*b*). Figure 6*c* plots one quantity against the other, revealing how when two successive stimuli are identical there is a benefit along both dimensions and ruling out speed-accuracy trade-offs. This also shows that serial dependence can lead to increased efficiency, not only for error, but also for the more conventional measure of reaction times.

## 4. Discussion

In this paper, we tested explicitly a model for response optimization that leverages on the previous stimulus to minimize response errors. The model was first developed to explain mapping number to space [2], adapted here for orientation reproduction. We show that serial dependence complies fully with the predictions of the Bayesian inference model. On one hand, we demonstrate that serial effects scale with stimulus uncertainty and similarity of current and previous stimuli; on the other, we show that both reproduction errors and reaction times are lower when serial dependence is strong, showing it is beneficial.

The first prediction of our model is that serial dependence scales with sensory uncertainty (figure 2*e*). We measured serial dependence and sensory discrimination thresholds for four types of Gabors varying in orientation and spatial frequency and found that they vary together. This result confirms our suggestion that oblique and cardinal stimuli have different serial effects, reflecting their different reliabilities [5,18,19,27]. The model simulated these effects well, using the measured estimates of noise, and hence no free parameters. This result extends our previous work on numerosity perception, where the model provided an excellent fit [2], and shows that it generalizes well to other perceptual tasks such as orientation reproduction. It suggests that stimulus uncertainty is the major driving effect of serial effects regardless of the source of noise.

We also replicate and model the tuning of serial dependence (figure 5) [3,4], an important feature as it proscribes integration of dissimilar stimuli, which could lead to large estimation errors. The model is not perfect, especially for large differences in orientations (around 60°), where it tends to predict more serial dependence than is actually observed. This may reflect inadequacies of the model, or the fact that the system does not have direct access to the model parameters (discussed below). However, the model does capture the fact that dissimilar features tend not to be integrated, a fact often observed in research into multisensory fusion: diverse stimuli are usually not integrated [28–30]. This points to stimulus similarity as a general perceptual rule, which is implemented in many systems and may be an important hallmark of the neural implementation of Bayesian processes. Interestingly our model gives some indication of appropriate parameters for measuring serial dependence. When the difference between stimuli is about 1 JND (*σ* = *d* in equation (3.7)), we expect the weight of the previous stimulus to be about 0.3.

Our model is an ideal observer model, developed to minimize total reproduction error (equation (3.5)). This results in a simple equation (equation (3.6)), where the theoretical weighting to the past depends on the reliability of past and present stimuli, and on their similarity. This is ideal. However, observers do not have access to the ground truth, either of the reliability of the stimuli or of the actual difference between them. All these parameters would need to be estimated in some way, and the estimation itself would not be noise-free. Several ideas have been advanced on how the system may extract an estimate of internal noisiness [31], and also of similarity [30], especially in multisensory research where this has long been acknowledged as a problem. Typically, researchers assume there is a ‘coupling prior’ [32], which they estimate from their data. We do not propose here any specific method of estimation, but rather use a theoretical value that should optimize performance. That this works well in predicting the data suggests that the system does have access to estimates of both noisiness and similarity of successive stimuli, although the mechanisms by which the parameters are estimated remain unknown and should be the subject of further research.

Importantly, serial dependence leads to an improvement of overall performance measured by response scatter (figure 5). This could only occur if noisiness were reduced by the integration of information over trails. Reduction of response scatter, as well as of overall response error (figures 5 and 6) was well predicted by our model and is in line with the Bayesian framework. Recently, a debate has emerged about whether serial dependence acts at the level of perception, or at the level of decision processes [3–6,33]. This distinction is fundamental in understanding the role of serial dependence for perception in general: if it acts only on decisional processes, it may have little to do with perception itself. The paradigms used here do not attempt to distinguish ‘perception’ from ‘decision’. Indeed, to model the data we measured perceptual reliability with a forced choice technique that emulated the conditions of the main experiments, including a 3 s pause between stimuli. Thus the measured noise includes not only sensory components of encoding the stimuli, but also effects that could reflect short-term memory, over the 3 s interval between trials. However, the fact that both data and model show very clear improvement in performance suggests that the serial dependence does not only bias perceptual decisions, but acts to improve perceptual efficiency, presumably by acting on perceptual processes themselves.

Our results also show that serial dependence can lead to decreases in reaction times. This is result was unexpected and shows that the error reduction is genuine and not simply the by-product of a speed-accuracy trade-off. Although the conditions of our experiment are far from ideal (because we did not explicitly request speeded responses), the results still suggest that serial dependence may facilitate the accrual of information over time for the reproduction task. This fact helps to relate the recent research line of serial dependence to the older priming literature, which has long documented speedup of responses when current presentations were primed by a suitable stimulus [34–37]. This is particularly obvious for examples when the term ‘priming’ has referred to low-level attentional selection [36,37], and has been conceived as a general perceptual process. Our demonstration that serial dependencies speed-up response times in a reproduction task show that biases in perception may well go hand in hand with perceptual distortions. After all, it is now becoming clear that the various processing stages of the brain accumulate evidence, and an alteration in lower-level representations that improves the quality of information also impacts on response speed.

## Data accessibility

Data used for this publication have been deposited into the Dryad Digital Repository: (http://dx.doi.org/10.5061/dryad.8ph33s0) [38].

## References

[1] Srinivasan MV, Laughlin SB, Dubs A 1982 Predictive coding: a fresh view of inhibition in the retina. Proc. R. Soc. Lond. B 216, 427–459. (10.1098/rspb.1982.0085) 6129637

[2] Cicchini GM, Anobile G, Burr DC 2014 Compressive mapping of number to space reflects dynamic encoding mechanisms, not static logarithmic transform. Proc. Natl Acad. Sci. USA 111, 7867–7872. (10.1073/pnas.1402785111) 24821771 PMC4040572

[3] Fischer J, Whitney D 2014 Serial dependence in visual perception. Nat. Neurosci. 17, 738–743. (10.1038/nn.3689) 24686785 PMC4012025

[4] Fritsche M, Mostert P, de Lange FP 2017 Opposite effects of recent history on perception and decision. Curr. Biol. 27, 590–595. (10.1016/j.cub.2017.01.006) 28162897

[5] Cicchini GM, Mikellidou K, Burr D 2017 Serial dependencies act directly on perception. J. Vis. 17, 6. 10.1167/17.14.6 29209696

[6] Bliss DP, Sun JJ, D'Esposito M 2017 Serial dependence is absent at the time of perception but increases in visual working memory. Sci. Rep. 7, 14739 (10.1038/s41598-017-15199-7) 29116132 PMC5677003

[7] Manassi M, Liberman A, Kosovicheva A, Zhang K, Whitney D 2018 Serial dependence in position occurs at the time of perception. Psychon. Bull. Rev. 1–9. (10.3758/s13423-018-1454-5) 29582377

[8] Liberman A, Fischer J, Whitney D 2014 Serial dependence in the perception of faces. Curr. Biol. 24, 2569–2574. (10.1016/j.cub.2014.09.025) 25283781 PMC4254333

[9] Taubert J, Alais D, Burr D 2016 Different coding strategies for the perception of stable and changeable facial attributes. Sci. Rep. 6, 32239 (10.1038/srep32239) 27582115 PMC5007489

[10] Alais D et al 2018 Eye gaze direction shows a positive serial dependency. J. Vis. 18, 11. 10.1167/18.4.11 29710301

[11] Taubert J, Van der Burg E, Alais D 2016 Love at second sight: sequential dependence of facial attractiveness in an on-line dating paradigm. Sci. Rep. 6, 22740 (10.1038/srep22740) 26986828 PMC4795074

[12] Alexi J, Cleary D, Dommisse K, Palermo R, Kloth N, Burr D, Bell J 2018 Past visual experiences weigh in on body size estimation. Sci. Rep. 8, 215 (10.1038/s41598-017-18418-3) 29317693 PMC5760712

[13] Manassi M et al 2017 The perceived stability of scenes: serial dependence in ensemble representations. Sci. Rep. 7, 1971 (10.1038/s41598-017-02201-5) 28512359 PMC5434007

[14] Suarez-Pinilla M, Seth AK, Roseboom W 2018 Serial dependence in the perception of visual variance. J. Vis. 18, 4. 10.1167/18.7.4 PMC6028984 29971350

[15] Rahnev D, Koizumi A, McCurdy LY, D'Esposito M, Lau H 2015 Confidence leak in perceptual decision making. Psychol. Sci. 26, 1664–1680. (10.1177/0956797615595037) 26408037 PMC4636919

[16] St John-Saaltink E, Kok P, Lau HC, de Lange FP 2016 Serial dependence in perceptual decisions is reflected in activity patterns in primary visual cortex. J. Neurosci. 36, 6186–6192. (10.1523/JNEUROSCI.4390-15.2016) 27277797 PMC6604889

[17] Dehaene S 2003 The neural basis of the Weber-Fechner law: a logarithmic mental number line. Trends Cogn. Sci. 7, 145–147. (10.1016/S1364-6613(03)00055-X) 12691758

[18] Mikellidou K et al 2015 The oblique effect is both allocentric and egocentric. J. Vis. 15, 24. 10.1167/15.8.24 PMC4909141 26129862

[19] Burr DC, Wijesundra SA 1991 Orientation discrimination depends on spatial frequency. Vision Res. 31, 1449–1452. (10.1016/0042-6989(91)90064-C) 1891831

[20] Brainard DH 1997 The psychophysics toolbox. Spat. Vis. 10, 433–436. (10.1163/156856897X00357) 9176952

[21] Alais D, Burr D 2004 The ventriloquist effect results from near-optimal bimodal integration. Curr. Biol. 14, 257–262. (10.1016/j.cub.2004.01.029) 14761661

[22] Cicchini GM, Arrighi R, Cecchetti L, Giusti M, Burr DC 2012 Optimal encoding of interval timing in expert percussionists. J. Neurosci. 32, 1056–1060. (10.1523/JNEUROSCI.3411-11.2012) 22262903 PMC6621155

[23] Ernst MO, Banks MS 2002 Humans integrate visual and haptic information in a statistically optimal fashion. Nature 415, 429–433. (10.1038/415429a) 11807554

[24] Jazayeri M, Shadlen MN 2010 Temporal context calibrates interval timing. Nat. Neurosci. 13, 1020–1026. (10.1038/nn.2590) 20581842 PMC2916084

[25] Kersten D, Mamassian P, Yuille A 2004 Object perception as Bayesian inference. Annu. Rev. Psychol. 55, 271–304. (10.1146/annurev.psych.55.090902.142005) 14744217

[26] Anobile G, Turi M, Cicchini GM, Burr DC 2012 The effects of cross-sensory attentional demand on subitizing and on mapping number onto space. Vision Res. 74, 102–109. (10.1016/j.visres.2012.06.005) 22727938

[27] Appelle S 1972 Perception and discrimination as a function of stimulus orientation: the ‘oblique effect’ in man and animals. Psychol. Bull. 78, 266–278. (10.1037/h0033117) 4562947

[28] Beierholm UR, Körding KP, Shams L, Ma WJ 2008 Comparing Bayesian models for multisensory cue combination without mandatory integration. In Advances in neural information processing systems 20, Proc. 2007 Conference.

[29] Bresciani JP, Dammeier F, Ernst MO 2006 Vision and touch are automatically integrated for the perception of sequences of events. J. Vis. 6, 554–564. 16881788 10.1167/6.5.2

[30] Roach NW, Heron J, McGraw PV 2006 Resolving multisensory conflict: a strategy for balancing the costs and benefits of audio-visual integration. Proc. R. Soc. B 273, 2159–2168. (10.1098/rspb.2006.3578) PMC1635528 16901835

[31] Ma WJ, Beck JM, Latham PE, Pouget A 2006 Bayesian inference with probabilistic population codes. Nat. Neurosci. 9, 1432–1438. (10.1038/nn1790) 17057707

[32] Ernst MO 2005 A Bayesian view on multimodal cue integration. In Human body perception from the inside out (eds Knoblich G et al.), pp. 105–131. New York, NY: Oxford University Press.

[33] Kiyonaga A, Scimeca JM, Bliss DP, Whitney D 2017 Serial dependence across perception, attention, and memory. Trends Cogn. Sci. 21, 493–497. (10.1016/j.tics.2017.04.011) 28549826 PMC5516910

[34] Kristjansson A, Campana G 2010 Where perception meets memory: a review of repetition priming in visual search tasks. Atten. Percept. Psychophys. 72, 5–18. (10.3758/APP.72.1.5) 20045875

[35] Forster KI, Davis C 1984 Repetition priming and frequency attenuation in lexical access. J. Exp. Psychol. Learn. Mem. Cogn. 10, 680–698. (10.1037/0278-7393.10.4.680)

[36] Maljkovic V, Nakayama K 1996 Priming of pop-out. II. The role of position. Percept. Psychophys. 58, 977–991. (10.3758/BF03206826) 8920835

[37] Maljkovic V, Nakayama K 1994 Priming of pop-out. I. Role of features. Mem. Cognit. 22, 657–672. (10.3758/BF03209251) 7808275

[38] Cicchini GM, Mikellidou K, Burr DC 2018 Data from: The functional role of serial dependence *Dryad Digital Repository*. (10.5061/dryad.8ph33s0) PMC6235035 30381379

